# Immunotargeting of Cancer Stem Cells

**DOI:** 10.3390/cancers15051608

**Published:** 2023-03-05

**Authors:** Ayse Sedef Köseer, Simona Di Gaetano, Claudia Arndt, Michael Bachmann, Anna Dubrovska

**Affiliations:** 1 National Center for Tumor Diseases (NCT), Partner Site Dresden: German Cancer Research Center (DKFZ), Heidelberg, Faculty of Medicine and University Hospital Carl Gustav Carus, Technische Universität Dresden, Helmholtz-Zentrum Dresden-Rossendorf (HZDR), 01307 Dresden, Germany; 2 OncoRay–National Center for Radiation Research in Oncology, Faculty of Medicine and University Hospital Carl Gustav Carus, Technische Universität Dresden, Helmholtz-Zentrum Dresden-Rossendorf (HZDR), 01309 Dresden, Germany; 3 Helmholtz-Zentrum Dresden-Rossendorf, Institute of Radiopharmaceutical Cancer Research, 01328 Dresden, Germany; 4 Mildred Scheel Early Career Center, Faculty of Medicine Carl Gustav Carus, Technische Universität Dresden, 01307 Dresden, Germany; 5 German Cancer Consortium (DKTK), Partner Site Dresden and German Cancer Research Center (DKFZ), 69120 Heidelberg, Germany; 6 Helmholtz-Zentrum Dresden-Rossendorf, Institute of Radiooncology-OncoRay, 01328 Dresden, Germany

**Keywords:** cancer stem cells, CSC, bsAB, CAR-T cells, cancer vaccines, immunotherapy

## Abstract

**Simple Summary:**

Tumor cells from the same specimen are functionally heterogeneous. Cancer stem cells (CSCs) are populations of tumor cells with self-renewal and differentiation properties. CSCs are found in nearly all solid and hematological tumors and are characterized by various surface or intracellular markers. These markers can be used to develop tumor-specific antibodies, cytotoxic immune cells, vaccines, and direct immune responses to the tumor cells, including CSC populations. This review discusses the emerging CSC-directed immunotherapies, the current state of their clinical development, the approaches to improve their safety and efficacy, and future strategies to strengthen anti-CSC immunotherapy.

**Abstract:**

The generally accepted view is that CSCs hijack the signaling pathways attributed to normal stem cells that regulate the self-renewal and differentiation processes. Therefore, the development of selective targeting strategies for CSC, although clinically meaningful, is associated with significant challenges because CSC and normal stem cells share many important signaling mechanisms for their maintenance and survival. Furthermore, the efficacy of this therapy is opposed by tumor heterogeneity and CSC plasticity. While there have been considerable efforts to target CSC populations by the chemical inhibition of the developmental pathways such as Notch, Hedgehog (Hh), and Wnt/β-catenin, noticeably fewer attempts were focused on the stimulation of the immune response by CSC-specific antigens, including cell-surface targets. Cancer immunotherapies are based on triggering the anti-tumor immune response by specific activation and targeted redirecting of immune cells toward tumor cells. This review is focused on CSC-directed immunotherapeutic approaches such as bispecific antibodies and antibody-drug candidates, CSC-targeted cellular immunotherapies, and immune-based vaccines. We discuss the strategies to improve the safety and efficacy of the different immunotherapeutic approaches and describe the current state of their clinical development.

## 1. Introduction

### 1.1. CSC Definition and Clinical Significance

Cancer remains a leading cause of death worldwide, despite advancements in its treatment [1]. Conventional cancer treatments such as surgery, chemotherapy, and radiotherapy may be the most effective during an earlier stage of tumor development. However, treatment efficacy might be limited by tumor genetic and epigenetic heterogeneity. Each tumor is composed of cells with different features, including therapy resistance, metastatic dissemination, differentiation potential, and potency to maintain tumor growth [2]. Cancer stem cells (CSC) are a population of tumor cells that sustain tumor growth and heterogeneity [3]. CSCs were first characterized by Dick and colleagues for acute myeloid leukemia (AML) and proven to possess two fundamental properties, such as the capacity of self-renewal (e.g., an asymmetrical division that produces an identical copy and more differentiated progeny cells) and differentiation into multiple cellular subtypes observed within tumors [4,5,6]. Because of their self-renewal and differentiation capabilities, it has been shown that leukemia-initiating stem cells could repopulate and induce AML in severe combined immunodeficient hosts (SCID) after transplantation [3]. Different studies supported the tumor-initiating and tumor-maintaining properties of CSCs in various tumor entities [7,8,9]. CSCs have been characterized by many surface or intracellular markers in solid and hematological tumors. The most used indicators for CSC identification are surface markers such as CD133, CD44, and CD123, as well as the activity of some intracellular proteins such as aldehyde dehydrogenase (ALDH), as recently reviewed [10,11,12,13,14].

As the tumor develops, the tumor microenvironment (TME) becomes progressively more crucial to maintaining the growth and functions of CSCs through the interplay with cellular components and modification of the extracellular matrix (ECM). The cellular components of TME, such as endothelial cells (ECs), mesenchymal cells (MSCs), immune cells, and cancer-associated fibroblasts (CAFs), play a role in therapeutic resistance by activating CSC-related signaling pathways such as Wnt, Notch, and nuclear factor kappa B (NF-κB) pathways [15,16,17,18]. In turn, CSCs, by secreting several signaling factors, including pro-inflammatory cytokines and chemokines, recruit and alter the functions of stromal and immune cells to facilitate tumor growth and progression, especially during and after anticancer treatments, hence compromising treatment outcomes [19]. The exosomes released from CSCs can form the premetastatic niche via upregulation of vascular endothelial growth factor (VEGF) and matrix metalloproteinase-2 (MMP-2), resulting in the activation of angiogenesis and promotion of metastatic growth [20].

Some CSCs can withstand conventional treatments such as chemo- and radiotherapy, which effectively destroys a large portion of the tumor bulk, causing tumor shrinkage. However, standard treatment often fails to prevent disease recurrence if the CSCs are not completely eradicated [21,22,23]. Some CSCs can resist the direct or indirect damages induced by ionizing irradiation. The described mechanisms of the CSCs radioresistance include the activation of DNA damage response mechanisms (e.g., ATM, ATR, and Chk1/2), the scavenging of reactive oxygen species (ROS), protection from oxidative stress, activation of the anti-apoptotic pathways, and residing in protective microenvironmental niches [6,23,24]. The chemotherapy-resistant CSCs might also exhibit enhanced expression and activity of the membrane transporters of the ATP-binding cassette (ABC) family, which are linked to multidrug resistance [17,22,23,24,25,26,27,28]. Due to their self-renewal and differentiation properties, some subpopulations of CSCs, termed metastasis-initiating cells, are capable of dissemination through the bloodstream and metastasis initiation in lymph nodes and distant organs [6,29]. Therefore, CSCs might serve as biomarkers for tumor diagnosis, prognosis, and therapy response prediction, whereas CSC-related markers can be utilized to develop more efficient targeted therapies.

### 1.2. CSC-Directed Therapeutic Approaches

Targeted therapies against CSCs are promising strategies to prevent cancer development and reduce the risk of recurrence [30]. Therefore, the signaling pathways regulating CSC maintenance and therapy resistance can be utilized as potential treatment targets. These pathways include, e.g., Hedgehog (Hh), Notch, JAK-STAT, PI3K/AKT/mTOR, Wnt/β-catenin, NF-κB, TGF-β, and FGF signaling [31,32,33,34,35,36,37,38,39,40,41]. Deregulation of these signaling pathways has been observed in various cancers [42,43,44,45,46]. Furthermore, these signaling axes interplay to regulate the self-renewal and differentiation properties of the CSCs, TME, and tumor development. Downstream transcription and pluripotency factors such as β-catenin, signal transducer, and activator of transcription 3 (STAT3), OCT4, Sox2, Nanog, KLF4, and MYC were also characterized as potential clinical targets [12,33,36,47,48,49,50,51]. A wide variety of inhibitors have been developed to specifically target these mechanisms, and there have been many clinical trials to test their anti-tumor activities [40,52]. However, CSC heterogeneity often impedes the efficacy of the therapeutic approaches against a single molecular target. The high dependency of normal stem cells on the pathways mentioned above might also explain normal tissue toxicity and side effects in some of these trials [53]. While conventional cancer therapies, such as radio- or chemotherapy, may eliminate the tumor bulk, treatment resistance of CSCs is suspected to be responsible for recurrence. Thus, it is critical to specifically target and destroy these cells to prevent or significantly delay tumor relapse. Immune cells infiltrating the tumor are a powerful natural mechanism to target and eradicate cancer cells. However, CSCs can create an immunosuppressive microenvironment through intrinsic and extrinsic mechanisms [54]. The immunosuppressive TME is produced by CSCs and other tumor and non-cancerous cells, such as CAFs and pro-tumor immune cells (i.e., regulatory T cells (Tregs), tumor-associated macrophages (TAMs), tumor-associated neutrophils (TANs), and myeloid-derived suppressor cells (MDCSs)) [55,56,57,58]. In addition, other extracellular physical and chemical factors of TME, such as pH and hypoxia, play a role in tumor immune evasion [59,60]. Tumor cells, including CSCs, can escape immune surveillance and immune-mediated cell killing by downregulating tumor-associated antigens (TAAs), increasing the expression of immune checkpoints such as programmed death-ligand 1 (PD-L1) and, therefore, inhibiting CD8+ cytotoxic T cells, and reducing the expression levels of major histocompatibility complex class I (MHC-I) and the transporter associated with antigen processing (TAP) molecules, which play vital roles in antigen processing and presentation processes [61,62,63,64,65,66,67,68,69,70,71,72,73]. Recently, it has been shown that AML stem cells suppress cytokine secretion and impair oxidative phosphorylation in T cells by overexpression of CD200 receptor [74]. It has also been demonstrated that PD-L1 was highly expressed on CD44^+^ CSCs compared to CD44^−^ non-CSCs in head and neck squamous cell carcinoma (HNSCC) [65] and regulates stemness in breast cancer [71]. CSCs, due to their plasticity, can also evade immunosurveillance by entering a dormant state or converting into quiescent cells, and by selective reduction of their immunogenic properties, whereas circulating tumor cells (CTCs), which share many properties with CSCs [6], are shielded from the cytotoxic activity of natural killer (NK) cells by TANs [75,76]. A high expression of MHC-I molecules on the normal autologous cells inhibits NK cell activation and function. Tumor cells often downregulate MHC-I and therefore reduce their recognition by CD8+ cytotoxic T cells. The MHC-I downregulation in tumors has been associated with unfavorable clinical prognoses [77]. However, some CSCs, e.g., in ovarian and renal cell carcinoma, upregulate MHC-I molecules on their surface that can potentially contribute to the NK cell regulation through MHC-I specific inhibitory receptors and CSC escaping NK cell-mediated cytotoxicity [78,79]. Therefore, activating the immune response targeting of CSCs via cancer vaccines, adoptive T and NK cell therapies, monoclonal antibodies, bispecific antibodies (bsABs), and immune checkpoint inhibitors (ICIs) can be a promising approach for achieving clinical success in the treatment of different cancer types.

## 2. Bispecific Antibodies and Antibody-Drug Conjugates

**Figure 1:**
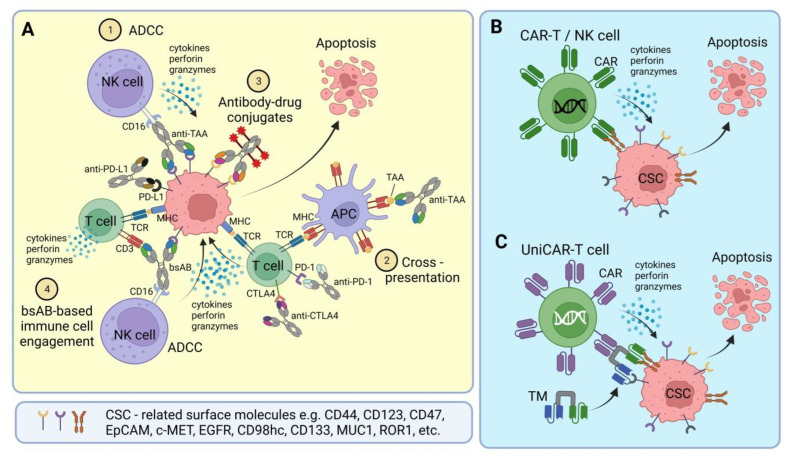
CSC-directed immunotherapeutic approaches. (**A**) Therapeutic antibody induces immune-mediated tumor cell killing by the induction of antibody-dependent cellular cytotoxicity (ADCC) (1). Antibody-mediated antigen delivery to the antigen-presenting cells (APC), such as dendritic cells (DCs), leads to effective T cell activation (2). T cells are also activated by inhibition of the immune checkpoint proteins: cytotoxic T-lymphocyte-associated protein 4 (CTLA-4), programmed cell death protein 1 (PD-1), and its ligand, PD-L1. Antibody-drug conjugates deliver potent chemotherapeutic agents to the target cells positive for a tumor-associated antigen (TAA) (3). Bispecific antibodies (bsAB) retarget immune cells to the TAA-expressing tumor cells (4). (**B**) CAR-T cells/CAR-NK cells are T or natural killer (NK) cells that are genetically modified with artificial chimeric antigen receptor (CAR) recruiting them to tumor cells. (**C**) Universal CAR (UniCAR) T cells recognize tumor cells via a short-lived target module (TM) which could be designed against more than one TAA. CAR—chimeric antigen receptor; CSC—cancer stem cells; MHC—major histocompatibility complex; TCR—T cell receptor. Created with BioRender.

The generally accepted view is that CSCs hijack the signaling pathways, attributed to normal stem cells, that regulate the self-renewal and differentiation processes. Therefore, the development of selective targeting strategies for CSCs, although clinically meaningful, is associated with significant challenges because CSCs and normal stem cells share many important signaling mechanisms for their maintenance and survival. Many chemical inhibitors used to target the CSC regulating signaling pathways described earlier, such as Notch, Hh, and Wnt/β-catenin signalings, also have a toxic effect on normal stem cells [80,81,82]. While there have been tremendous preclinical efforts to target CSC populations from inside the cell, considerably less effort has focused on the cell-surface targets. Cancer immunotherapies are based on inducing the anti-tumor immune response by specific activation and targeted redirecting of immune cells toward tumor cells. Since the first characterization of CSC phenotype in AML by Dick and colleagues in the late 1990s, various surface markers of CSC in hematopoietic malignancies and solid tumors have been identified, including CD44, CD133, CD117, CD123, CD47, CD98hc, and others [4,5,6]. These surface proteins play a pivotal role in the CSC interaction with their niche, cell–cell communication, nutrient uptake, and regulation of the immune system, and provide specific targets for CSC-directed immunotherapies (Figure 1).

In 1993, Seiter and colleagues published a seminal study describing the anti-tumor effect of anti-CD44v antibody using preclinical syngeneic rat xenograft models [83]. The first monoclonal antibody (mAb), rituximab, a chimeric IgG1 against the B-cell-specific antigen CD20 highly expressed on non-Hodgkin’s lymphoma (NHL) cells, was approved for treatment of NHL in 1997 [84] paving the way for immunotherapeutic applications in oncological diseases. Shortly after, the clinical studies in patients with solid tumors showed the therapeutic potential and safety of radioimmunotherapy with the (186)Re-labeled humanized mAb bivatuzumab, which as directed against a CSC-related protein, the CD44 isoform variant 6 (CD44v6) (Table 1). The transmembrane glycoproteins of the CD44 family are highly expressed in the different types of malignant cells, including CSCs, and contribute to tumorigenesis by regulating various cell surface receptors [85]. This family includes several variant isoforms, and CD44v6 is one of the best-studied isoforms serving as a co-receptor for the receptor tyrosine kinases (RTKs), such as vascular endothelial growth factor receptor-2 (VEGFR-2), epidermal growth factor receptor (EGFR), and mesenchymal-epithelial transition factor (c-Met) [85,86]. Unfortunately, early clinical testing of bivatuzumab merstansine, a CD44v6-specific antibody conjugated with an antimicrotubule agent, in 2006 led to a fatal outcome related to the binding of the anti-CD44v6 antibody to skin keratinocytes and associated severe skin toxicity [87]. Therefore, further development of the CD44-targeted drug conjugate was discontinued. A newer anti-CD44 recombinant humanized antibody, RG7356, showed an acceptable safety profile in a clinical trial for patients with advanced solid tumors; however the clinical efficacy was modest. The trial was terminated early as no dose–response relationship was reported [88]. However, in patients with AML, RG7356 induced differentiation of CD34^+^ leukemic stem-like cells and accumulation of CD68^+^ macrophages [89].

Integrin associated protein (IAP), or CD47, is another promising target overexpressed on cancer cells, including CSCs, in different types of hematological and solid tumors. CD47 plays a critical role in regulating the homeostasis of immune cells (e.g., T cells, macrophages, and dendritic cells (DCs)), including their activation, differentiation, migration, and death. It is also crucial for tumor growth, as CD47 expression on solid tumors results in evasion of the innate immune response [90,91]. CD47 exerts these activities by interacting with integrin receptors, e.g., α_v_β_3_, and activating integrin-dependent signaling molecules such as focal adhesion kinase (FAK) [92]. CD47 also binds to a transmembrane glycoprotein, signal regulatory protein α (SIRPα), therefore activating protein tyrosine phosphatases such as Src homology 2 (SH2) domain-containing phosphatase-1 (SHP-1) and SHP-2 [91]. In addition, CD47 also acts as a receptor for thrombospondin 1 (TSP-1) [93]. Of importance, previous studies using syngeneic prostate tumor models in CD47 deficient mice showed that inhibition of CD47 might have opposite consequences for tumor growth depending on the target cells: CD47/TSP-1 inhibition in tumor stromal ECs induces angiogenesis and tumor progression, and decreased TSP1 production in tumors from CD47 deficient mice reduced macrophage recruitments, whereas blocking the binding of CD47 on tumor cells with SIRPα on macrophages and DCs might, in contrast, induce an antitumor immune response and reduce tumor growth [94]. Breast cancer progression is associated with the development of intratumoral hypoxia and activation of hypoxia-inducible transcriptional factors (HIFs). HIF1 was shown to stimulate the CD47 expression in breast CSCs, enabling them to avoid macrophage-mediated phagocytosis. In addition, CD47 positively regulates breast CSC phenotypes and properties [95]. Furthermore, inhibition of CD47 by anti-CD47 antibodies was shown to effectively target pancreatic CSCs by increasing macrophage-mediated immunity and tumor cell apoptosis [96]. Of importance, CD47 expression and, therefore, immune evasion in ovarian CSCs has been shown to be induced by surrounding bulk tumor cells [97]. Recent studies also demonstrated that CD47 transcription is regulated via HER2–NF-κB pathway, and antibody-mediated blockage of both CD47 and HER2 synergized with radiotherapy for the treatment of syngeneic mouse breast tumors. Notably, radiotherapy increased the rate of macrophage-mediated phagocytosis in the tumors treated with anti-CD47 antibodies in combination with Herceptin or single anti-CD47 antibodies compared to radiotherapy applied alone in the orthotopic breast tumor models. This study showed synergistic tumor inhibition by a combination of radiotherapy plus anti-CD47 and anti-HER2 immunotherapy [98]. IBI188, also known as Letaplimab, is a recombinant human anti-CD47 mAb. IBI188, in combination with azacytidine (AZA), a DNA methyltransferase inhibitor, showed promising efficacy and a manageable toxicity profile in patients with newly diagnosed higher risk myelodysplastic syndrome (MDS) (NCT04485065) [99]. IBI188 is currently being tested in several early-phase clinical trials in combination with AZA in patients with AML (NCT04485052) and as a single treatment in patients with advanced solid cancers and lymphomas (NCT03717103 and NCT03763149).

Another attractive target is the interleukin-3 receptor (IL-3R) alpha chain, or CD123, which is highly expressed in the undifferentiated precursor (blast) cells, but present at a low level on normal hematopoietic stem cells, and is associated with the development of AML, acute lymphoblastic leukemia (ALL), B-lymphoid leukemia (BLL), hairy cell leukemia (HCL), Hodgkin lymphoma, blastic plasmacytoid dendritic neoplasms (BPDCN), and MDS [100,101]. The early clinical trials for the recombinant IL-3 fused with diphtheria toxin (SL-401, or tagraxofusp) targeting CD123 showed clinical effectiveness, especially in patients with BPDCN [102,103]. Based on these clinical studies, tagraxofusp was approved by the Food and Drug Administration (FDA) for the treatment of patients with BPDCN cancer [102].

These clinical investigations fueled the further development of the anti-CD123 treatment using immunological approaches. In particular, a humanized anti-CD123 mAb Talacotuzumab (JNJ-56022473, CSL362) was engineered to have increased affinity for CD16 on NK cells and target CD123 positive cells through NK cell-mediated antibody-dependent cellular cytotoxicity (ADCC) [104]. The preclinical studies demonstrated an efficient depletion of CD123^+^ blasts in samples from AML patients [104] and in AML xenograft models that was mediated by both allogeneic and autologous NK cells [105,106]. However, in the clinical analysis as a single agent in elderly high-risk MDS or AML patients, Talacotuzumab demonstrated significant toxicities that resulted in early treatment discontinuation as well as in limited clinical efficacy, explained by the alteration in the NK- and T cell repertoire in these patients before treatment start [105]. Furthermore, Talacotuzumab was tested for its safety and efficacy in combination with decitabine, a chemotherapy drug, and in comparison with decitabine alone in patients with AML. These clinical studies showed no improvement of clinical efficacy for combination treatment compared to the single treatment with decitabine [107].

To improve the clinical effectiveness of the CD123-targeted antibody therapy, an antibody-drug conjugate was developed. To this end, a humanized high-affinity anti-CD123 antibody was linked to the DNA-alkylating cytotoxic compound from the class of indolinobenzodiazepine pseudodimer (IGN) [108]. This anti-CD123-targeting antibody-drug conjugate, called IMGN632, demonstrated robust antitumor efficacy in preclinical models for different hematological malignancies, including AML [108] and BPDCN [109] xenograft models, while sparing normal bone marrow cells, which express low levels of CD123. The early clinical study of IMGN632, given as monotherapy or in combination with AZA and venetoclax (VEN), a Bcl-2 inhibitor, in patients with CD123-positive AML, showed a manageable safety profile. The administration of IMGN632 was associated with a high objective response rate (ORR) of 75% and composite complete remission rate (CCR) of 40% in the high-intensity cohort of patients with AML, whereas ORR/CCR rates were even higher in the cohort of VEN-naïve patients (100%/60%, respectively) [110].

In contrast to the conventional monospecific antibody, bsAB are artificially engineered antibodies with dual specificity for different epitopes attributed to the same or different antigens, therefore offering a variety of therapeutic opportunities, including retargeting of immune cells and modulations of the ligand and receptor action with an efficacy that is hard to achieve for single antibodies [111]. The application of bsAB in cancer treatment is a fast growing area of clinical research. The first clinically approved bsAB, Catumaxomab, targeting the T cell antigen CD3 and human epithelial cell adhesion molecule (EpCAM), was designed to recruit T cells to tumors [112]. Since then, bsABs are one of the most promising tools for targeting tumor malignancies through the recruitment of immune cells such as T cells or NK cells (cell-bridging bsAB) or by antigen cross-linking [113]. In the laboratory, a bsAB is generated by genetic engineering, chemical conjugation of two purified monoclonal antibodies, or by quadromas, in which fused hybridoma cells produce bsABs along with non-functional by-products [114]. Recombinant bsABs might have different designs described in detail elsewhere [111,115,116]. The T cell engaging approach with cell-bridging bsAB was used to develop several CSC-related bsABs, which bind to the patient’s T cells through CD3/T cell receptor (TCR), CD28, or other surface molecules mediating T cell activation and proliferation. On the other hand, they bind to target antigens on tumor cells. Thus, T cell engaging bsABs specifically redirect T cells to target-positive tumor cells. The antigen cross-linking with bsAB can be used for simultaneous blocking and inhibition of two targets on the surface of tumor cells, including CSCs, thereby preventing ligand-induced activation and tumor growth [113]. Simultaneous bsAB binding to the checkpoint regulators on the surface of T cells e.g., PD-L1 and CTLA-4, might potentiate anti-tumor immune response. The design of novel bsAB enables binding to multiple targets, therefore making them a multi-specific antibody (MsAb) [115,117]. Table 1 includes several antibody-based CSC-targeted therapies that have already entered clinical trials. These therapies, in particular, include CD123 × CD3 bsAB, whose commercial name is Flotetuzumab (MGD006). Flotetuzumab has been evaluated in phase I/II clinical trials for refractory AML, and has shown encouraging anti-leukemic activity and acceptable safety. In particular, complete remission was observed in 26.7% of patients with refractory AML [118].

Several CD47-targeted bsABs are currently being tested in early-phase clinical trials, including HX009, PD-1 × CD47 bsAB, tested in patients with relapsed or refractory lymphoma (NCT05189093). HX009 binds to PD-1 expressed on T cells and CD47 on tumor cells. By this, HX009 blocks the binding of CD47 on tumor cells with SIRPα on macrophages and DCs and, therefore, activates macrophage-mediated phagocytosis of the CD47-expressing tumor cells. On the other hand, the binding of HX009 to PD-1 prevents the interaction between PD-1 and its ligands, PD-L1 and PD-L2, and inhibits the downstream signaling pathways. This signal inhibition recovers effector T cell functions and activates cytotoxic T cell-mediated antitumor immunity [119]. Preliminary results suggest that HX009 is well-tolerated and shows strong antitumor activity [120,121]. Another CD47 targeting bsAB, IBI322, with a dual specificity for CD47 and PD-L1 [122], is also being tested in several clinical trials in patients with advanced malignant tumors (NCT04328831, NCT04912466, NCT04338659) and hematologic malignancies (NCT04795128), but no clinical data has been reported yet. In addition, bsAB targeting CD47 and the B-lymphocyte antigens CD20 (IMM0306) or CD19 (TG-1801) also entered clinical testing for patients with B-cell lymphoma or chronic lymphocytic leukemia (CLL), (NCT04806035, NCT03804996) and B-cell NHL (NCT04746131).

Another example is Amivantamab, a bsAB targeting EGFR and c-Met driving tumor growth in patients with non-small cell lung cancer (NSCLC). NSCLC progression is frequently associated with activating mutations in the kinase domain of EGFR. Some of these mutations, such as in-frame base pair insertions in exon 20 (ex20-ins), result in tumor resistance to conventional EGFR tyrosine kinase inhibitors [123]. EGFR and another receptor tyrosine-protein kinase, c-Met, cooperatively regulate tumor cell proliferation, migration, and activation of the downstream signaling pathways. Because of the synergy between the EGFR and c-Met pathways, their dual inhibition is critical for the treatment of NSCLC [124]. Amivantamab bsAB inhibits the activation of both receptors by binding to their extracellular domains, preventing ligand-induced activation and triggering receptor degradation. In addition, it activates tumor destruction by effector immune cells through Fc-mediated mechanisms, such as ADCC [125]. Amivantamab was demonstrated to be efficient against NSCLC with a resistance mutation in EGFR and c-Met activation [125]. Clinical trials (e.g., CHRYSALIS; NCT02609776; and others) have shown acceptable toxicity and anti-tumor efficacy of Amivantamab in patients with locally advanced or metastatic NSCLC and EGFR Ex20ins mutations based on the ORR of about 40% and duration of response [126]. In 2021, Amivantamab was approved by the US FDA for the treatment of patients with advanced or metastatic NSCLC with EGFR ex20-ins mutations, whose disease had progressed during or after platinum-based chemotherapy [127]. Of importance, c-Met has been characterized as a regulator of CSC populations in different types of solid tumors, including pancreatic cancer [128,129], prostate cancer [130], and colorectal cancer [131]. A novel bsAB c-Met × CTLA-4 targeting c-Met and CTLA-4, a negative regulator of T cell activation, showed significant anti-tumor activity in lung cancer models in vitro and in vivo. This anti-tumor effect was at least partially mediated by the inhibition of the CD166^+^ positive lung CSC populations [131].

EpCAM is another marker highly expressed in tumor cells, including CSCs [132]. Catumaxomab (Removab) is a EpCAM × CD3 bsAB approved in the European Union in April 2009 for the treatment of malignant ascites, a condition developing in patients with different types of epithelial cancers [112]. It is called a trifunctional antibody (trAb) due to its ability to bind tumor cells, T cells, and accessory cells (e.g., macrophages, DCs, and NK cells) through its intact Fc region [133]. The results from clinical studies (NCT00836654) demonstrated that the application of catumaxomab is associated with the depletion of CD133^+^/EpCAM^+^ CSCs from malignant ascites in patients with the ovarian, pancreatic, and gastric cancer [134].

Antibody-based cancer immunotherapy, particularly monospecific and bsAB therapy, is a promising strategy for cancer treatment [113], and most bsABs are still in the early phase of clinical trials. However, some biological and clinical factors might compromise the efficacy of bsAB and limit their clinical translation. In particular, the application of bsAB, like some other types of immunotherapy, e.g., chimeric antigen receptor (CAR) T cells, might cause potentially fatal adverse effects such as cytokine release syndrome (CRS)—systemic inflammatory response associated with high cytokine levels in peripheral blood [135]. There is a hope that many bsABs, currently being tested in more than 300 clinical trials, will be introduced in clinical practice [136]. However, only one bsAB for treating solid malignancies, Amivantamab, an EGFR/c-Met specific bsAB for the treatment of patients with NSCLC, has been clinically approved [137]. Limitations that might impact the efficacy of the therapeutic antibodies for the treatment of solid tumors include poor tumor penetration, unequal distribution, and endocytic clearance in tumor cells [138]. In addition, some tumor cell populations, such as CSCs, can occupy hypoxic niches where antibody delivery is complicated due to the sparse presence of blood vessels [23,139]. Notably, a robust assay to measure CSC functions in tumor samples, besides surface marker expression, is yet missing. Given the heterogeneity and plasticity of CSC, developing these assays would be essential for the reliable evaluation of CSC-directed immunotherapy in clinical trials [40]. The loss of the tumor antigen, which serves as bsAB's target and consequent tumor resistance and immune escape, is an additional complication for the clinical application of bsAB [140,141]. Furthermore, no single surface marker is currently available to define the entire CSC population in a given tumor entity, or even in one individual tumor [142]. There is evidence of high variability of the CSC phenotype between patients with a given type of cancer and a high heterogeneity of CSC within one individual tumor. Mutation changes, epigenetic reprogramming, and microenvironmental stimuli induce CSC plasticity during the treatment, cancer progression, and upon relapse [142,143,144]. An essential challenge is the scarcity of tumor-specific antigens that are not present in normal tissues: more than 70% of known CSC surface markers appear on normal adult and embryonic stem cells [145]. Even low epitope expression on normal cells can be associated with severe normal tissue toxicities from immune therapy [146]. Therefore, improving treatment specificity by inventing new cancer-specific treatment targets and sequential or combinational targeting of two or more tumor antigens during the course of treatment could reduce normal tissue toxicity and overcome tumor antigen escape. Thus, developing multispecific antibodies [147] and multispecific CAR T cell immunotherapy (as described in the next chapter) could be a promising strategy to improve treatment options for patients with malignant tumors.

**Table 1:** Selected clinical trials for antibody-based therapies targeting tumor cells, including CSCs.

Specificity/Generic Name	Description	Tumor Entity Tested	Clinical Trials/Approvals	References
CD44v6/Bivatuzumab (BIWA 4)	mAb against CD44v6, (186)Re-labeled	Inoperable recurrent and/or metastatic HNSCC, NSCLC, breast cancer	Phase I: NCT02204059, NCT02204046, NCT02254018 Outcome: Antitumor effects and effective tumor targeting was observed. Administration is well tolerated.	[148,149]
CD44v6/Bivatuzumab—mertansine	mAb against CD44v6, conjugated mertansine	Incurable HNSCC or esophagus squamous cell carcinoma (ESCC), recurrent or metastatic breast cancer	Phase I: NCT02254044, NCT02254031, NCT02254005, NCT02254018 Outcome: one fatal drug-related adverse skin event had occurred. Further clinical development was discontinued.	[87,150,151,152]
CD44v6/RG7356	mAb against CD44v6	Advanced CD44-expressing solid malignancies.	Phase I study: NCT01358903 Outcome: acceptable safety profile, modest clinical efficacy was observed. The study was terminated due to the absence of a clinical and pharmacodynamic dose-response relationship	[88]
CD44v6/RG7356	mAb against CD44v6	AML	Phase I study; NCT01641250 Outcome: the treatment was generally safe and well tolerated. Out of 44 patients, two patients achieved complete or partial response and one patient had stable disease.	[89]
CD123/JNJ-56022473 /Talacotuzumab	7G3 mAb against CD123	Elderly high-risk MDS or AML failing hypomethylating agents	Phase II: NCT02992860 Talacotuzumab as a single agent; Outcome: limited clinical efficacy and significant toxicity	[153]
CD123/JNJ-56022473 /Talacotuzumab	7G3 mAb against CD123	CD123-positive AML	Phase II/III study: NCT02472145 Talacotuzumab in combination with decitabine versus decitabine alone; Outcome: no improvement in efficacy versus decitabine alone	[107]
CD123/IMGN632	mAb G4723A against CD123 conjugated with DNA-alkylating payload of the IGN cytotoxic compounds	CD123-positive AML	Phase Ib/II study; NCT03386513 IMGN632 is given as monotherapy or in combination with AZA and/or VEN; Outcome: manageable toxicity profile; high ORR (of 75%) and CCR (of 40%) in high intensity cohort; ORR/CCR rates were even higher in the cohort of VEN-naïve patients (100%/60%, respectively)	[110,154]
CD47 IBI188/Letaplimab	mAb against CD47	Newly diagnosed higher risk MDS	Phase I study: NCT04485065 The preliminary results suggest that IBI188 in combination with AZA showed a promising efficacy and a manageable toxicity profile	[99]
CD123 and CD3 Flotetuzumab/ MGD006	bsAB (CD3ε × CD123)	Relapsed/refractory AML	Phase I/II study: NCT02152956 Outcome: acceptable safety profile, encouraging anti-leukemic activity (the complete remission rate (CRR)/CRR with partial hematological recovery was 26.7%; an overall response rate was 30.0%	[118]
CD47 and PD-1 HX009	bsAB antibody binding CD47 and PD-1	Relapsed or refractory lymphoma	Phase I/II study: NCT0409776, The preliminary results suggest that HX009 is well-tolerated and showed strong antitumor activity	[120,121]
EGFR and c-MET Amivantamab/Rybrevant/ JNJ-61186372	bsAB antibody binding EGFR with one Fab and c-Met with the other Fab	Advanced or metastatic solid tumors including EGFR-mutated NSCLC	Amivantamab was approved by the US FDA for the treatment of patients with advanced or metastatic NSCLC with EGFR ex20ins mutations, whose disease has progressed on or after platinum-based chemotherapy.	[125,126,127]
EpCAM and CD3 Catumaxomab/Removab	EpCAM × CD3; trAb binding tumor cells, T cells, and accessory cells (e.g., macrophages, DC, and NK cells through its intact Fc region	Malignant ascites derived from epithelial tumors	Catumaxomab was approved in the European Union in April 2009 for the treatment of malignant ascites, but was withdrawn in 2017 for commercial reasons.	[112]

## 3. CSC-Targeted Immune Cells

In contrast to the antibody-based immunotherapy available off-the-shelf, adoptive cell therapy (ACT) has been developed as more individualized anti-cancer immunotherapy when cytotoxic lymphocytes, such as T cells or NK cells, are customized for each patient. The cytotoxic activities of T cells and NK cells are mediated by releasing the pore-forming protein perforin, granzyme serine protease, and pro-inflammatory cytokines, making them attractive candidates for the ACT [155,156,157]. The objective regression of cancer after ACT was first documented in 1988 for patients with metastatic melanoma treated with autologous tumor-infiltrating lymphocytes [158]. The promising results of the ACT applications stimulated the genetic engineering of immune cells to improve tumor-specific response and to broaden ACT application to other types of cancer. For this, T cells were genetically modified using viral vectors to overexpress either conventional TCRs or artificial CAR. T cells equipped with TCRs recognize the antigens presented by MHC molecules on the surface of tumor cells, whereas T cells with CARs recognize tumor-specific cell surface antigens that do not need to be restricted by MHC [159].

The idea for CAR design was coined by Gross and colleagues in 1989 [160]. The CAR structure contains four major modules: (I) the extracellular domain responsible for the antigen binding and made by the variable domains of the heavy (VH) and light (VL) chains of tumor-specific immunoglobulins, (II) the spacer domain connecting the extracellular domain to the transmembrane domain, (III) the transmembrane domain, and (IV) the cytoplasmic domain derived from activating immune receptors, e.g., TCR. The initial design of CARs included a single intracellular CD3ζ motif. These CARs were able to efficiently trigger the signal for the activation and effector function of T cells against target antigen-expressing cells [160]. However, the early phase clinical trials for ovarian cancer patients showed the lack of patient antitumor response associated with a short-term persistence of genetically modified T cells in the blood of patients and poor T cell trafficking to the tumor sites [161]. The next generations of CARs included additional activation motives from costimulatory molecules, such as CD28 [162,163] and 4-1BB/CD137 [164,165], in the intracellular part of CAR molecules. This design is associated with robust T cell proliferation and full activation, better persistence in vivo, and amelioration of T cell exhaustion. The selection of the optimal CAR design is a fast-expanding field of immunology, including specificity, affinity and avidity of antigen binding regions, spacer length, and transmembrane domain interactions, as well as type, number, and order of the costimulatory domains [166]. The design of CAR constructs for NK cells includes similar components. However, the unique characteristics of NK cells motivated researchers to develop CARs containing NKG2D immunoreceptor elements and additional signaling subunits such as DAP10, DAP12 [167,168,169].

The manufacturing of CAR-T cells consists of multiple steps, including the collection of peripheral blood mononuclear cells (PBMCs) from the patient, T cell isolation, activation, genetic manipulation for CAR expression, expansion, and quality checks for further applications [170]. CAR-T cell-based immunotherapy is currently being investigated in more than 800 clinical trials [171]. Due to impressive clinical success rates, several autologous CAR-T based anti-cancer treatments have been clinically approved [172]. Kymriah (Tisagenlecleucel) was the first anti-CD19 CAR-T therapy approved by the FDA in 2017 for relapsed or refractory pediatric and young-adult B-cell ALL, and then for adult relapsed or refractory diffuse large B-cell lymphoma [173]. Since then, four additional anti-CD19 CAR-T-based anti-cancer therapies and one anti-B-cell maturation antigen (BCMA) CAR T product have been approved for the treatment of B-cell lymphomas/leukemias and multiple myeloma [172,174,175], respectively. Analysis of the ongoing clinical trials evaluating CAR-T cells revealed that CD19 and BCMA are the most frequently used antigens for CAR-T therapies against hematological malignancies, whereas, for solid tumors, CAR-T cells are directed against, e.g., mesothelin, carcinoembryonic antigen (CEA), Mucin 1 (MUC1), HER2, EGFR, and glypican-3 (GPC3) [171]. Among these targets, MUC1 was characterized as a stemness driver in colorectal cancer where MUC1 forms a complex with MYC transcriptional factor and activates the expression of leucine-rich repeat-containing G protein-coupled receptor 5 (LGR5) gene, a marker of the intestine stem cells and CSCs [176]. In addition, MUC1 also activates the expression of other CSC markers, such as ALDH1, BMI1, and the pluripotency factors Oct4, Nanog, and Sox2171 [177]. MUC1 induces tumor cell plasticity and epigenetic reprogramming by coupling MYC activation with activation of other transcription factors such as STAT3, NF-κB, and E2F [178]. Furthermore, several CSC-targeted CAR-based therapies entered early-stage clinical trials. These clinical trials include CAR-T cells targeting CD44v6 in stomach cancer lymphosarcoma, AML, and multiple myeloma; CD133 in relapsed and/or chemotherapy refractory advanced malignancies; c-Met in patients with melanoma and breast carcinoma [179], EpCAM in nasopharyngeal carcinoma, breast cancer, gastric cancer and other EpCAM positive solid tumors (Table 2).

Although several CAR-based therapies for blood cancers have been clinically successful, CAR-modified immune cells still face several hurdles in clinical application for solid tumors. Different biological factors might decrease CAR-T cell efficacy, including a loss of tumor antigen (antigen escape) associated with the development of therapy resistance, lack of antigen specificity resulting in the “on-target off-tumor” toxicity against normal tissues, CAR-T exhaustion [180], immunosuppressive tumor microenvironment, and poor CAR-T cell trafficking and tumor infiltration [181]. Furthermore, like other immunotherapy types, such as antibody-based cancer treatment, CAR-T cell-based therapy can cause severe side effects, including the above-described CRS and immune effector cell-associated neurotoxicity syndrome (ICANS) [182]. In addition, the ex vivo manipulation of T cells for the autologous CAR-T cell production is mainly performed as a manual or semi-automated process resulting in considerable variability and high acquisition costs [183].

**Figure 2:**
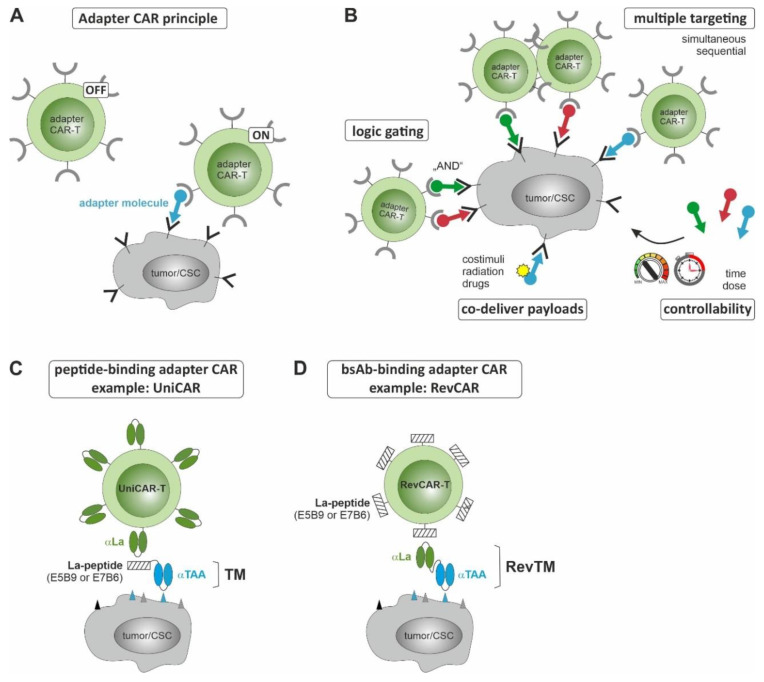
Adapter CAR approaches. (**A**) Adapter CAR T cells do not recognize a surface antigen. Thus, they are inactive (“OFF”). CAR T cells are activated for tumor cell killing (“ON”) only in the presence of a crosslinking adapter molecule. (**B**) Due to the modular design, adapter CAR T cell activity can be controlled via the adapter molecule (dosing, timing). Simultaneous or sequential application of different adapter molecules enables multiple and logic-gated tumor targeting. In the adapter CAR system, additional payloads can be co-delivered via adapter molecules to enhance anti-tumor responses. (**C**) In the UniCAR system, as an example of peptide-binding adapter CARs, CAR T cells are redirected for tumor cell killing via a so-called target module (TM) that is composed of a tumor-specific binding arm and a short La-peptide epitope (E5B9 or E7B6) recognized by the UniCAR. (**D**) In the RevCAR system, bispecific antibodies (bsAB), termed reversed target modules (RevTMs), mediate RevCAR T cell/tumor cell interactions and, thus, tumor cell killing. RevTMs simultaneously recognize a tumor antigen and the La-peptide epitope (E5B9 or E7B6) used to construct the RevCAR.

The growing awareness of these limitations has driven the development of new generations of CAR T cells, and various strategies are being pursued to overcome the existing difficulties. Bispecific CARs, multi-CARs, and logic-gated CAR T cells are being developed to enhance the specificity as well as efficiency, and thereby reduce off-tumor effects of CAR T cell products [184,185,186]. The incorporation of suicide switches [187,188] and “biodegradable” CAR T cells [189,190] are further attempts to particularly address the safety concerns of CAR T cell therapy. Besides these and many other strategies, modular adapter CAR platforms currently represent a rapidly and steadily growing field to create safer and more efficient CAR T cell retargeting strategies [191]. The main concept of this technology is to separate the effector and targeting functions of conventional CARs (Figure 2A). Thus, adaptor CAR T cells are designed to be redirected for tumor cell killing only in combination with a second tumor-specific component. Unlike conventional CAR-T cells, they do not recognize any surface antigen and are, therefore, switched off by default. For cross-linking with target cells, and thus activation, a so-called adapter molecule is required. In principle, it consists of a tumor-specific binding site and an interaction site for CAR-T cell recruitment. The modular concept of adapter CAR approaches allows (I) control of therapy-related side effects by adaptor molecule dosing, (II) highly flexible targeting of different tumor-associated antigens, either simultaneously or sequentially, thereby increasing treatment specificity/efficacy and lowering the risk of tumor escape and off-tumor effects, and (III) co-delivery of payloads to locally enhance anti-tumor effects [191] (Figure 2B). The interaction of adapter CAR T cells and the adapter molecules is based on different connection systems following two major concepts: (I) adapter CARs recognizing diverse tags incorporated into the adapter molecule, e.g., peptide tags [192,193,194], FITC [195], biotin [196], dinitrophenyl [197], and (II) adapter CARs redirected to tumor cells via bispecific antibodies [198,199,200,201,202]. The interaction between human La/SS-B peptide epitopes (E5B9, E7B6) [203,204] and the corresponding anti-La antibody binding domains [205], for example, led to the development of both the peptide-binding adaptor CAR “UniCAR” [206] (Figure 2C) and the corresponding bsAB-binding adaptor CAR “RevCAR” [201] (Figure 2D). Under physiological conditions, naturally occurring human La/SS-B resides in the nucleus and is inaccessible to unwanted interactions with UniCAR/RevCAR components, rendering the corresponding adapter CAR-T cells inactive. The UniCAR is a second-generation CAR constructed by fusing an anti-La single-chain fragment variable (scFv) as an extracellular binding domain to the transmembrane and intracellular domain of CD28, as well as the signaling domains of CD3zeta (Figure 2C). UniCAR T cells can be cross-linked with tumor cells and induce tumor cell lysis only in the presence of a tumor-specific target module (TM). Such TMs are designed by connecting a tumor-specific binding moiety, e.g., peptide ligands [207], nanobodies [208], or antibody-derived fragments [209,210,211,212], to the La-epitopes. Vice versa, in the RevCAR system, La-peptide epitopes are used as the extracellular domain of the RevCARs (Figure 2D) [201,213]. Thus, bsABs (termed RevTMs) simultaneously target the La epitope and a tumor-associated antigen. They are utilized to bridge CAR T cells and tumor cells and, thereby engaging RevCAR T cells for efficient tumor cell lysis. Other recent designs have further focused on developing adaptor CARs that recognize common features of already approved drugs, e.g., the P329G Fc mutation of therapeutic antibodies [214] or a binding pocket within the Fab arm of monoclonal antibodies [215]. In preclinical in vitro and in vivo studies, adapter CAR T cells have been successfully redirected against various CSC-related antigens, such as CD123 [213,216,217], EpCAM [196,200,218] and CD98hc [219]. By modifying T cells with two different RevCARs and fine-tuning the selected adapter molecules, the RevCAR system was successfully applied for dual “AND”-gate targeting of CD33 and CD123 on AML blasts, highlighting the versatility of the platform technology (Figure 2B) [213]. Such logic-gated approaches will allow more specific targeting of tumor cells, including CSCs, thereby reducing unwanted toxicities against healthy tissues. More recently, CD123-directed UniCAR T cells showed the first proof-of-concept for functionality and controllability in a phase I clinical trial with AML patients [220,221]. So far, treatment was completed in 12 patients and proved to be tolerable, with overall mild adverse effects and only one dose-limiting toxicity. The observed treatment-related side effects, e.g., myelosuppression, disappeared rapidly when the infusion of the adapter molecules was interrupted. After the patients had recovered, therapy could be resumed by repeated TM administration. Ten patients treated with UniCAR-T-CD123 therapy have shown a clinical response, including two complete remissions with incomplete count recovery and four partial responses [220,221].

In contrast to CAR-T cells, CAR-NK cells are less likely to induce off-tumor toxicities and adverse side effects, such as CRS and neurotoxicity, that could be partially attributed to their short lifespan in the bloodstream and different types of secreted cytokines [222]. The preclinical and clinical studies demonstrated that allogenic NK cells have a low risk for graft versus host disease (GVHD) [223,224,225], can be prepared from different sources (e.g., cord blood, haploidentical donors, induced pluripotent stem cells, iPSC) [224,225,226,227] and expanded ex vivo for “off-the-shelf” allogeneic applications [228]. Unlike CAR-T cells, CAR-NK cells kill tumor cells through CAR-mediated and CAR-independent mechanisms. Due to these unique properties, several clinical trials are exploiting CAR-NK cells’ cytotoxic activity targeting specific tumor antigens in patients with hematopoietic malignancies and solid tumors [222]. These clinical trials include targeting the CSC-related antigens, such as CAR-NK cells, against CD123 in AML and MUC1 in solid tumors (Table 2). Although CAR-NK therapy emerged as a more cost-efficient and safer immunotherapy than CAR-T cells, it is challenged by the short in vivo lifespan and limited proliferation capacity of NK cells that compromise long-lasting therapeutic responses. Similar to CAR-T cells, CAR-NK anti-cancer efficiency is reduced by tumor heterogeneity, immune suppressive microenvironment, off-tumor cell killing, and poor CAR-NK infiltration into solid tumors [229].

**Table 2:** Selected clinical trials for immune cell-based therapies targeting CSC-related tumor antigens.

Specificity /Generic Name	Description	Tumor Entity Tested	Clinical Trials	References
CD44v6	CAR-T cells	CD44v6 positive stomach cancer, lymphosarcoma	Phase I/II; NCT04427449 No results were posted.	
CD44v6	MLM-CAR44.1 T cells; CD44v6 CAR-T cells were genetically modified to express herpes simplex virus (HSV)-TK Mut2 suicide gene to minimize toxicity	AML, Multiple Myeloma	Phase I/II; NCT04097301 Outcome: terminated due to the inability to close the study in a clinically relevant time frame.	[230]
CD133	CAR-T cells	Relapsed and/or chemotherapy refractory advanced malignancies (liver cancer, pancreatic cancer, brain tumor, breast cancer, ovarian tumor, colorectal cancer, acute myeloid and lymphoid leukemia)	Phase I/II; NCT02541370 Outcome: out of 21 enrolled patients, 1 had a partial response, 14 had stable disease during 2–16.3 months, and 6 progressed after treatment start; hyperbilirubinemia was the most common high-grade adverse event	[231]
CD38-CART/ CD33-CART/ CD56-CART/ CD123-CART/ CD117-CART/ CD133-CART/ CD34-CART/ MUCl-CART	single CAR-T or double CAR-T cells with CD33,CD38, CD56,CD123, CD117,CD133, CD34 or MUCl	AML	Phase: n/a; NCT03473457 Outcome: terminated because the therapeutic effect was not as expected. No results were posted	
EpCAM	CAR-T cells	Nasopharyngeal carcinoma, breast cancer, gastric cancer and other EpCAM positive solid tumors	Phase I; NCT02915445 No results were posted	
EpCAM- and TM4SF1	CAR-T cells	Refractory/recurrent advanced pancreatic cancer, colorectal cancer, gastric cancer or lung cancer	Phase: n/a; NCT04151186 No results were posted	
CD123	CAR-NK cells	AML	Phase: I; NCT05574608 No results were posted	
MUC1	CAR-NK cells	MUC1 positive relapsed or refractory solid tumor	Phase I/II; NCT02839954 No results were posted	
CD123	Preconditioning (lymphodepletion) with cyclophosphamide and fludarabine followed by treatment with UniCAR-T and CD123 TM	Relapsed/refractory AML	Phase I; NCT04230265 Outcome: the initial results suggest that the treatment is well tolerated with mild adverse effects; out of three treated patients, one patient had a partial remission and two patients had complete remission with incomplete hematologic recovery	[220]

## 4. Cancer Stem Cell Vaccines

**Figure 3:**
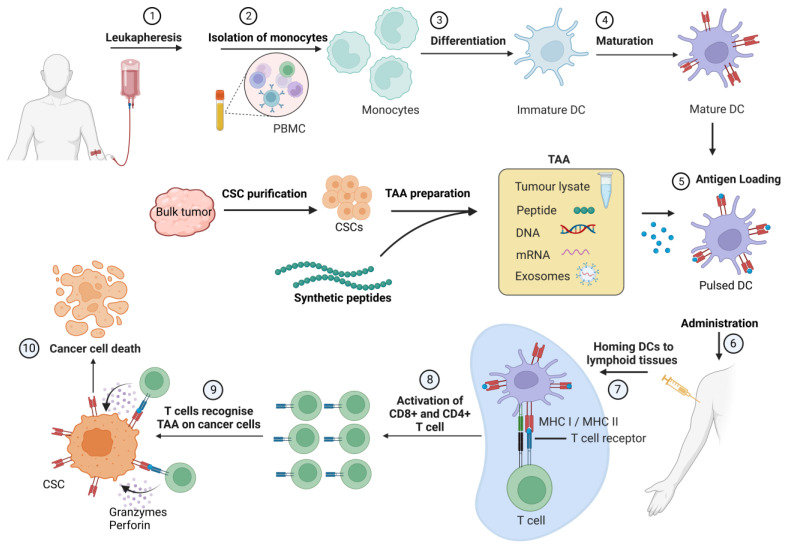
Development of the DC autologous vaccines. The DC vaccines are most commonly prepared by ex vivo differentiation of the autologous monocytes into immature DCs. After maturation, DCs are pulsed with cancer antigens and administered back to the patient, where they activate antigen-specific T cells. CSC—cancer stem cells; MHC I and MHC II—major histocompatibility complex class I and class II; PBMC—peripheral blood mononuclear cells; TAA—tumor-associated antigen. Created with BioRender.

An additional promising approach to boost immune responses against CSCs is to use CSCs as a source of antigens to pulse antigen-presenting DCs and develop anti-CSC DC vaccines. DCs were first discovered in 1973 by Ralph Steinmann and Zanvil A. Cohn as a phagocytic cell population in the murine spleen [232]. DCs are an immune cell population bridging innate and adaptive immune responses. They present the processed epitopes to CD4+ T cells and CD8+ T cells through MHC II and MHC I, respectively, and secrete cytokines critical for the survival and proliferation of T cells, NK cells, and T cell tumor infiltration [233]. DC vaccination is a promising form of immunotherapy, and many DC vaccines have been developed in the past years and tested in clinical trials. The DC vaccines are most commonly prepared by ex vivo differentiation of the autologous precursor cells into immature DCs, the maturation of DCs by addition of a cytokine cocktail, and then pulsing them with cancer antigens in the form of antigen peptides, tumor cell lysates, exosomes, or mRNAs. Afterwards, mature DCs are administered back into the patients where they activate antigen-specific T cells [231] (Figure 3). Numerous clinical studies have shown that DC vaccinations are both safe and efficient anti-cancer therapies capable of inducing an immunological response, increasing tumor-infiltrating lymphocytes, and improving overall survival (OS) [231,234].

Dendritic CSC vaccination (CSC-DC) is a promising form of DC-mediated immunotherapy. The administration of DCs loaded with MUC1-derived peptide, alone or in combination with other tumor-specific antigens, has been tested in several clinical trials for patients with refractory NSCLC [235], pancreatic cancer [236,237,238], biliary cancer [238], and castration-resistant prostate cancer (CRPC) [239]. These studies showed that the vaccine was well tolerated and associated with clinical responses.

Another approach employing the peptide-loaded DCs is the ALDH peptide-based DC vaccine [240,241]. ALDH is a family of metabolic enzymes responsible for the detoxification of intracellular aldehydes through their oxidation to the carboxylic acids [242]. A high level of ALDH activity measured by the ALDEFLUOR analysis is used as a marker to isolate CSC populations in a wide variety of solid malignancies, such as breast cancer, prostate cancer, lung cancer, colon cancer, sarcoma, and HNSCC [240,242,243]. The ALDH family includes 19 genes. Out of them, several ALDH isoforms are highly expressed in CSCs, including ALDH1A1 and ALDH1A3 [242]. In addition to being markers for CSC, both genes play critical functional roles in the regulation of the activation of the retinoic acid signaling, PI3K/AKT pathway, ethanol and amino acid metabolism, and cell defense against ROS [242,243,244]. The critical role of ALDH proteins in tumor development and therapy resistance makes them promising therapeutic targets. In the first adoptive therapy experiments for targeting ALDH-positive CSCs, ALDH1A1-specific CD8+ T cells were induced in vitro by the DCs pulsed with ALDH1 [88,89,90,91,92,93,94,95,96] peptide and injected intravenously into the xenograft-bearing immunodeficient mice [241]. This study demonstrated that ALDH1 peptide-specific CD8+ T cells inhibited primary tumors growing subcutaneously and lung metastases [241].

Similar to other immunotherapy directed against a single antigen, the efficacy of the peptide-based DC vaccines could be compromised by heterogeneity and plasticity of antigen expression in tumor cells, including CSCs. A pulsing of DCs with the entire tumor cell lysate, potentially including the whole repertoire of the tumor antigens, is another promising strategy for developing CSC-specific DC vaccines. The tumor cells with high ALDH activity (ALDH^high^) can be identified and isolated quickly by fluorescence-activated cell sorting (FACS), providing a source of antigens for developing CSC-targeted therapeutic approaches. Ning and coauthors first confirmed high tumorigenicity of ALDH^high^ cell populations compared to ALDH^negative^ cells using murine melanoma D5 and squamous cell carcinoma SCC7 syngeneic xenograft tumor models in the immunocompetent C3H and C57BL/6 mice. Next, they evaluated the antitumor immunity induced by vaccination with murine bone-marrow-derived DCs pulsed with the lysate of ALDH^high^ cells (CSC-tumor pulsed DC, CSC-TPDC) compared with the lysate of whole unsorted heterogeneous tumor cells (H-TPDC). They demonstrated that the vaccination of DCs pulsed with the lysate of ALDH^high^ cells induced significantly higher protective immunity against tumors than H-TPDCs, as well as DCs pulsed with the lysate of ALDH^negative^ cells [245]. An additional approach to increase anti-tumor immunogenicity by targeting multiple epitopes is a pulsing of DCs with mRNA derived from CSCs. A recent in vitro study by Sumransub et al. demonstrated a preclinical efficacy of an mRNA-based DC vaccine using patient-derived breast cancer cells. In this study, DCs were differentiated from PBMCs of a healthy donor and pulsed with mRNA isolated from CD44^+^/CD24^−^ CSC population. This finding revealed that CSC mRNA induces a more potent cytotoxic T cell response as compared to mRNA isolated from the entire tumor cell population [72].

The DC vaccine based on the CSC lysates was also recently tested in a clinical trial for newly diagnosed or recurrent glioblastoma (GBM). In particular, the phase I clinical trial assessed the safety and tolerability of the autologous DC vaccine pulsed with lysate derived from allogeneic GBM stem-like cells. The glioblastoma cells used for the vaccine preparation were isolated from a single patient and propagated as neurospheres in serum-free conditions for CSC enrichment. For the autologous vaccine preparation, PBMCs were collected by leukapheresis and used to isolate monocytes, which were differentiated to DCs and pulsed with GBM CSC lysates. DC vaccines were administered intradermally. The therapy was safe and well tolerated. A subset of the patients (9 of the assessed 25 patients) developed a cytotoxic T cell immune response. The study was not powered to evaluate treatment efficacy, although a comparison of the progression-free survival (PFS) and OS with historical control suggested that CSC-targeted immunotherapy can be a treatment of patients with GBM [246].

Results of the clinical studies for DC-based vaccines demonstrated that their combination with therapies that stimulate immune responses and inhibit immune suppression might be more effective for cancer treatment than vaccine administration alone [238]. ICI represents one of the most used treatments in the last decade. It targets immune checkpoint molecules, including CTLA-4, PD-1, and PD-L1, and thereby enhances the immune response to cancer [234]. As discussed in chapter 1.2, CSCs can contribute to tumor immune evasion. Recent studies revealed that PD-L1 promotes the expression of stemness markers [65,70,71,72] and CSC populations with a high level of PD-L1 expression might be associated with tumor immune evasion. To overcome these therapeutic challenges, several studies combined CSC-DC vaccines and ICIs. For example, Hassani Najafabadi et al. developed a nanoparticle vaccine system to deliver ALDH1A1 and ALDH1A3 epitope peptides to antigen-presenting cells (APCs) in vivo and induce T cell responses against ALDH-high CSCs [247]. According to the study, vaccination with high-density lipoprotein nanodisks (ND) loaded with ALDH epitopes reduced the frequency of ALDH^high^ CSCs in tumor tissue when combined with anti-PD-L1 therapy, and it exerted strong inhibitory effects on tumor growth in the syngeneic D5 melanoma and 4T1 breast cancer models [247]. Liao et al. used the synthetic ALDH1A1, and ALDHA1A3 peptides and DCs derived from murine bone marrow for the preparation of the CSC-targeted DC vaccines. DCs were administered subcutaneously into a C57BL/6 mice tumor model bearing syngeneic D5 murine melanoma. This study demonstrated that the DC vaccine induced T cell proliferation, humoral immune response, and T cell cytotoxicity against ALDH^high^ tumor cells, and inhibited D5 tumor growth in vivo. The dual ALDH1A1 and ALDH1A3 peptide DC vaccine possessed significantly higher antitumor activity as compared to the single ALDH1A1 or ALDH1A3 peptide vaccines [240]. Of importance, this study also proved that anti-PD-L1 treatment significantly enhanced the number of CD3+ tumor-infiltrating lymphocytes and antitumor effect of this vaccine [240]. Zheng et al. investigated the CSC targeting effect of the CSC-DC vaccine combined with a dual blockade of immune checkpoints, such as PD-L1 and CTLA-4. This study confirmed the efficacy of the ALDH^high^-DC vaccines for the treatment of the syngeneic melanoma B16-F10 tumors growing in the immunocompetent C57BL/6 mice. Importantly, this study revealed that animals treated with the dual blockade of PD-L1 and CTLA-4 and CSC-DC vaccine conferred significantly more tumor regression than the CSC-DC vaccine alone. They also showed that the combination of the CSC-DC vaccine and immune checkpoint blockade significantly induced CD8+ T cell proliferation and CSC-specific cytotoxic T cell activity compared with the CSC-DC vaccine alone. This study provided the scientific basis for a clinical trial involving the combination of the CSC-DC vaccine and simultaneous PD-L1 and CTLA-4 blockades for improved tumor control in patients with cancer [248].

A combination of CSC-targeted DC vaccines with conventional therapy is a promising approach to target both CSC and non-CSC cell subsets and to prevent the interconversion between the cell populations. Lu et al. evaluated the therapeutic efficiency of ALDH^high^-CSC lysate-pulsed DCs in combination with local tumor radiation therapy (RT) given in 6 doses of 8.5 Gy. They employed syngeneic D5 melanoma and squamous sarcoma SCC7 tumor model in immunocompetent C57BL/6 and C3H mice. This study confirmed the therapeutic efficacy of the CSC-DC vaccine and demonstrated that it has a higher anti-cancer efficiency in the adjuvant setting when administered after RT. The studies conducted by Hu et al. also assessed the therapeutic potential of CSC-DC vaccination in adjuvant setting [249]. They revealed that ALDH^high^-DC treatment after surgical tumor resection significantly reduced local recurrence, prevented lung metastases, and reduced tumor ALDH^high^ CSC populations in the immunocompetent C3H mice bearing syngeneic squamous carcinoma SCC7 tumors. The study of El-Ashmawy et al. suggested that the efficacy of the cisplatin-based chemotherapy for the treatment of murine Ehrlich carcinoma can be improved by its combination with the DC vaccine developed against the CD44^+^/CD24^−^ CSC-like cell population [250].

Taken together, the immune targeting of CSCs represents a promising approach to cancer treatment. Furthermore, its combination with ICIs and conventional therapy such as surgery and chemo- and radiotherapy could be a strategy to optimize its therapeutic effectiveness.

## 5. Preclinical and Clinical Trials of Combination Therapies with Immunotherapy and Conventional Therapies Targeting CSC Markers

Conventional treatment strategies for patients with malignant diseases include surgery, chemotherapy, and radiotherapy, referred to as the traditional “three pillars” of cancer treatment. Combining immunotherapies with conventional treatments has recently become a cornerstone of cancer therapy (Table 3). It has been reported that many conventional cancer treatments, such as radiotherapy and chemotherapy, have additional immune activation mechanisms of action, including the depletion of immunosuppressive Tregs and MDSCs. The therapy-induced cell death releases tumor antigens recognized, processed, and presented to T lymphocytes by APCs [251,252,253,254,255]. Many studies have depicted the significance of combination therapy for the treatment of CSCs. In preclinical studies using an AML model, a combination of a DNA methylation inhibitor AZA and CD47 blockade via 5F9 mAb have resulted in increased macrophage-mediated phagocytosis in vitro compared to single treatments, inhibited AML growth, and prolonged survival in xenograft mice models [251]. Following these findings, a phase 1b study has demonstrated that the combination of magrolimab, a well-tolerated humanized anti-CD47 antibody, with AZA showed a greater ORR compared to AZA treatment alone, with a more rapid response time in AML patients ([252], NCT03248479). Bone marrow analysis has depicted significantly lower levels of leukemic stem cells in responding patients treated with the combination treatment. In another preclinical study, the combination of targeting receptor tyrosine kinase-like orphan receptor 1 (ROR1)-dependent signaling, which is associated with CSC maintenance and self-renewal, via cirmtuzumab and ibrutinib, which blocks B-cell receptor signaling, was more effective than single-agent treatments in reducing the number of CLL cells in the spleens of immunodeficient mice [253]. These preclinical findings have provided a rationale for clinical studies. A phase 1/2 trial is currently testing this combination in CLL patients, showing encouraging complete response results compared to ibrutinib treatment alone, suggesting a synergistic effect of the therapy ([254], NCT03088878). There are also preclinical studies that test the combination of immunotherapy with radiotherapies. Sequential treatment with fractionated photon irradiation followed by CD98hc-directed UniCAR treatment has exhibited a synergistic cytotoxic effect on radioresistant HNSCC spheroids compared to single treatments, underlining an improved antitumor effect [246]. A phase 2 clinical trial composed of induction treatment with FOLFOXIRI (folinic acid, 5-fluorouracil, oxaliplatin, and irinotecan) drug combination and bevacizumab, a mAb targeting angiogenic factor VEGF, followed by chemoradiotherapy and bevacizumab treatment, has been conducted in patients with advanced and resectable rectal adenocarcinoma. Still, no results have been posted on the ClinicalTrials.gov website yet (NCT03085992). Of importance, small-molecule inhibitors of fat mass and obesity-associated protein (FTO) demonstrated to suppress AML stem cell self-renewal as well as inhibit immune checkpoint expression, and immune evasion, hinting at the broad potential of anti-CSC therapy [255]. The CSC model predicts that therapies targeting the tumor bulk may induce tumor shrinkage; however, the responses will not be durable. Accumulating evidence suggests that stemness is rather a transient feature, and the de-differentiation of the non-CSC populations can replenish the pool of CSCs. Therefore, combining conventional therapies and CSC-targeting treatment could be a more efficient therapeutic strategy to improve therapeutic efficacy.

## 6. Conclusions

The therapeutic success of anti-cancer immunotherapy depends on the ability of the immune system to detect and destroy tumors as foreign tissue. However, the efficiency of cancer immunotherapy can be limited by immune evasion. CSCs possess multiple mechanisms to escape immune surveillance and create an immunosuppressive microenvironment. Results of the preclinical studies demonstrated that a combination of CSC-targeted immunotherapies with an immune checkpoint blockade stimulates anti-tumor immune responses and might be a more promising cancer therapy for further clinical studies. Tumor cell plasticity and heterogeneity also reduce the efficacy of anti-CSC therapy. Developing immunotherapy against more than one CSC antigen might lower the risk of tumor escape and non-specific toxicity. A combination of CSC-specific immunotherapy with conventional treatments, such as radio- and chemotherapy targeting the bulk tumor cells and ICI enhancing the anti-tumor immune response, could be a strategy to prevent CSC replenishment by non-CSC de-differentiation. Furthermore, conventional therapy such as radiotherapy and chemotherapy can activate multiple immune-stimulating mechanisms, highlighting the consideration for their combination with anti-CSC immunotherapy [266,267,268,269,270]. The development of preclinical models that can recapitulate human immunity and heterogeneous CSC populations in human tumors is another challenge for developing efficient and clinically relevant CSC-targeted treatment. These demands for improving the translational potential of preclinical immunology models are currently addressed using humanized [271], naturalized [272], syngeneic [273], and genetically engineered mice models [274]. These models better recapitulate the human immune system compared to conventional human tumor xenografts and can be used for precise monitoring of immune–tumor interaction [275] and for hypothesis-driven experimentation [274]. In addition, different alternative strategies to modeling immunity are being further refined, including ex vivo cultures preserving the human tissue structures, microfluidic devices, and 3D engineered tissues providing physiologically relevant microenvironments [276]. The development of robust CSC analysis in the patient-derived specimens during treatment is essential to assess the efficacy of the CSC-targeted immunotherapy [40]. It is important to notice that most of the above-described clinical trials are developed to target the bulk tumor cells and do not specifically aim to eliminate CSCs. Although several above-described immunotherapies are showing promising clinical efficacy, including considerable CRR, for example, for the patients with AML treated with IMGN632 and Flotetuzumab, the analyses of bulk tumor response for evaluating the treatment efficacy might not be suitable indicators for its specificity against CSCs. Therefore, CSC-related assays are crucial to assessing the clinical response in these studies, including analyses of CSC frequency and characterization of their self-renewal and tumor-initiating properties. The preclinical efforts to improve the effectiveness of CSC-targeting approaches, including bsAB, CARs, and vaccines, and the data obtained from ongoing clinical trials, might pave the road for CSC-directed treatments to become a clinical reality.

## Figures and Tables

**Table 3:** Preclinical/clinical trials of combination therapies targeting CSC markers.

Preclinical Studies					
Experimental Model	Target	Immunotherapy	Combined Therapy	Cancer Type	Refs
In vitro (cell line)	CD47	5F9 mAb	AZA (cytotoxic analogue of the nucleoside cytidine, inhibitor of DNA methyltransferase)	AML	[251]
In vivo (cell-line derived xenograft)					
In vivo (patient-derived xenograft)	ROR1	Cirmtuzumab	Ibrutinib (Bruton’s tyrosine kinase (BTK) inhibitor)	CLL	[253]
In vitro (cell line)	EGFR	CAR NK-92	Cabozantinib (VEGFR-2 inhibitor)	Renal cell carcinoma	[114]
In vivo (cell-line derived xenograft)					
In vitro (cell line)	Carbonic Anhydrase IX (CAIX)	CAR T	Sunitinib (multi-targeted receptor kinase inhibitor)		[131]
In vivo (cell-line derived xenograft)					
In vitro (cell line)	HER2	HER2 CAR NK-92	Apatinib (VEGFR-2 inhibitor)	Gastric cancer	[256]
In vivo (cell-line derived xenograft)					
In vitro (cell line)	IL-6	Tocilizumab	MK-0752 (γ-secretase inhibitor)	Breast cancer	[257]
In vivo (cell-line derived xenograft)					
In vivo (patient-derived xenograft)					
In vivo (cell-line derived xenograft)	EpCAM	CAR-NK-92	Regorafenib (multitargeted kinase inhibitor)	Colorectal cancer	[258]
In vivo (syngeneic models)	ALDH^High^ CSCs	ALDH^High^-DC vaccine	Anti PD-L1 antibody	D5 murine melanoma and 4T1 murine breast cancer	[247]
In vivo (syngeneic models)	ALDH^High^ CSCs	ALDH^High^-DC vaccine	Anti PD-L1 antibody; Anti CTLA-4 antibody	B16-F10 murine melanoma tumors	[248]
In vivo (cell-line derived xenograft)	CD133	CAR-T	Cisplatin (DNA-binding cytotoxic drug)	Gastric cancer	[259]
In vitro (Cell line)		CAR NK-92		Ovarian cancer	[260]
In vivo (cell-line derived xenograft)	CD44^+^/CD24^−^ CSCs	CD44+/CD24− CSC-pulsed DC vaccine		Ehrlich carcinoma	[250]
In vitro (patient-derived cell line)	OAcGD2	8B6 mAb	Temozolomide (alkylating agent)	GBM	[261]
In vivo (Patient-derived xenograft)					
In vivo (cell-line derived xenograft)	CD105	TRC105	Conventional fractionated RT (5 × 2 Gy)	Prostate cancer	[262]
In vivo (Cell-line derived xenograft)	CD47	Anti-CD47 mAb	Conventional fractionated RT (1 × 5 Gy; 4 × 5 Gy; 1 × 10 Gy)	Small cell lung cancer, colon cancer	[263]
In vitro (3D model)	CD98	UniCAR T + CD98 TM	Conventional fractionated RT (2 × 2 Gy)	HNSCC	[246]
In vivo (Cell-line derived xenograft)	CD25	Anti-CD25 mAb	Conventional fractionated RT		[264]
Clinical trials					
NCT number	Target	Immunotherapy	Combined therapy	Cancer type	Refs
NCT03248479	CD47	Magrolimab (Hu5F9-G4)	AZA	AML/MDS	[252]
NCT03088878	ROR1	Cirmtuzumab	Ibrutinib	CLL	[254]
NCT02259582	DLL4	Demcizumab	Carboplatin + pemetrexed	Non-squamous NSCLC (DENALI)	[265]

## Abbreviations

ABC	ATP-binding cassette
ACT	Adoptive cell therapy
ADCC	Antibody-dependent cellular cytotoxicity
ALDH	Aldehyde dehydrogenase
ALDH^high^	High ALDH activity
ALL	Acute lymphoblastic leukemia
AML	Acute myeloid leukemia
APC	Antigen-presenting cell
AZA	Azacitidine
BCMA	B-cell maturation antigen
BLL	B-lymphoid leukemia
BPDCN	Blastic plasmacytoid dendritic neoplasm
bsAB	Bispecific antibody
BTK	Bruton’s tyrosine kinase
CAF	Cancer-associated fibroblast
CAR	Chimeric antigen receptor
CCR	Composite complete remission rate
CD44v6	CD44 isoform variant 6
CEA	Carcinoembryonic antigen
CLL	Chronic lymphocytic leukemia
CRPC	Castration-resistant prostate cancer
CRR	Complete remission rate
CRS	Cytokine release syndrome
CSC	Cancer stem cell
CSC-DC	Dendritic CSC vaccination
CSC-TPDC	CSC-tumor pulsed DC
CTLA-4	Cytotoxic T-lymphocyte-associated protein 4
CTC	Circulating tumor cell
DC	Dendritic cell
EC	Endothelial cell
ECM	Extracellular matrix
EGFR	Epidermal growth factor receptor
EpCAM	Epithelial cell adhesion molecule
ESCC	Esophagus squamous cell carcinoma
ex20-ins	Insertions in exon 20
FACS	Fluorescence-activated cell sorting
FAK	Focal adhesion kinase
FDA	Food and Drug Administration
FOLFOXIRI	Folinic acid, 5-fluorouracil, oxaliplatin and irinotecan
FTO	Fat mass and obesity-associated protein
GPC3	Glypican-3
GVHD	Graft versus host disease
H-TPDC	Heterogenous-tumor pulsed DC
HCL	Hairy cell leukemia
Hh	Hedgehog
HIF	Hypoxia-inducible transcriptional factor
HNSCC	Head and neck squamous cell carcinoma
ICANS	Immune effector cell-associated neurotoxicity syndrome
ICI	Immune checkpoint inhibitor
IGN	Indolinobenzodiazepine pseudodimer
IL-3R	Interleukin-3 receptor
iPSC	Induced pluripotent stem cell
LGR5	Leucine-rich repeat-containing G protein-coupled receptor 5
mAb	Monoclonal antibody
MDCS	Myeloid-derived suppressor cell
MDS	Myelodysplastic syndrome
MET	Mesenchymal-epithelial transition
MHC-I	Major histocompatibility complex class I
MMP-2	Matrix metalloproteinase-2
MsAb	Multi-specific antibody
MSC	Mesenchymal cells
MUC1	Mucin 1
ND	Nanodisk
NF-κB	Nuclear factor kappa B
NHL	Non-Hodgkin’s lymphoma
NK	Natural killer cell
NSCLC	Non-small cell lung cancer
ORR	Objective response rate
OS	Overall survival
PBMC	Peripheral blood mononuclear cell
PD-1	Programmed cell death protein 1
PD-L1	Programmed death-ligand 1
PFS	Progression-free survival
ROR1	Receptor tyrosine kinase-like orphan receptor 1
ROS	Reactive oxygen species
RT	Radiation therapy
RTK	Receptor tyrosine kinase
scFv	Single-chain variable region
SCID	Severe combined immunodeficient
SH2	Src homology 2
SHP-1	Src homology 2 domain-containing phosphatase-1
SHP-2	Src homology 2 domain-containing phosphatase-2
SIRPα	Signal regulatory protein α
STAT3	Signal transducer and activator of transcription 3
TAA	Tumor-associated antigen
TAM	Tumor-associated macrophage
TAP	The transporter associated with antigen processing
TAN	Tumor-associated neutrophil
TCR	T cell receptor
TM	Targeting module
TME	Tumor microenvironment
Treg	Regulatory T cell
trAb	Trifunctional antibody
TSP-1	Thrombospondin 1
UniCAR	Universal CAR
VEGF	Vascular endothelial growth factor
VEGFR-2	Vascular endothelial growth factor receptor-2
VEN	Venetoclax
VH	Heavy chain
VL	Light chain
